# Tiryāq in traditional Persian medicine: a survey of antidotal plants and their modern pharmacological potential

**DOI:** 10.3389/fphar.2025.1503149

**Published:** 2025-04-02

**Authors:** Haniye Mirhosseini, Majid Dadmehr, Bahareh Sadat Yousefsani, Farhad Seif, Fatemeh Eghbalian

**Affiliations:** ^1^ Institute for Studies in Medical History, Persian and Complementary Medicine, Iran University of Medical Sciences, Tehran, Iran; ^2^ Department of Traditional Medicine, School of Persian Medicine, Iran University of Medical Sciences, Tehran, Iran; ^3^ Student Research Committee, Iran University of Medical Sciences, Tehran, Iran; ^4^ Department of Traditional Pharmacy, School of Persian Medicine, Iran University of Medical Sciences, Tehran, Iran; ^5^ Department of Photodynamic, Medical Laser Research Center, Yara Institute, Academic Center for Education, Culture, and Research (ACECR), Tehran, Iran

**Keywords:** antidote, tiryāq, medicinal plants, Persian medicine, detoxification

## Abstract

**Purpose:**

Tiryāq (Theriac) refers to a single or compound medication historically utilized as a general antidote against numerous poisons in several ethnomedical traditions, especially in traditional Persian medicine (PM). This study aims to summarize the traditional uses, phytochemistry, and pharmacological activities of medicinal plants with tiryāq properties, with a particular focus on their anti-hepatotoxic, hepatoprotective, neuroprotective, and cardioprotective activities.

**Methods:**

Classical texts of traditional PM were broadly reviewed to extract information about tiryāq and its mechanisms. In addition, a detailed search of scientific databases was performed to validate the pharmacological properties of plants traditionally recognized for their antidotal effects.

**Results:**

Thirty-one medicinal plants with antidote properties were identified. The primary function of tiryāq, as described in PM, is to neutralize toxins and bolster the immune system. These plants have cardiotonic, hepatoprotective, and neuroprotective properties. In addition to their antidotal applications, tiryāq remedies were traditionally used to manage chronic cough, stomachache, asthma, colic, and other ailments. Modern pharmacological studies support these applications, highlighting the plants’ antiviral, immunomodulatory, and antioxidant properties, especially against acute respiratory viral infections and other inflammatory circumstances.

**Conclusion:**

Tiryāq plays a pivotal role in fortifying essential organs, including the heart, brain, and liver. Its prophylactic use during epidemics, along with its antioxidant and immune-stimulating properties, underscores its therapeutic potential. Further research is needed to conclusively determine the efficacy and broader therapeutic applications of medicinal plants with tiryāq properties.

## 1 Introduction

Poisoning is one of the common medical emergencies and represents an important global public health challenge. It comprises the most prevalent causes of morbidity and mortality, which is the second largest reason for morbidity worldwide (Alnasser et al., 2022). Harmful substances and toxins in the air, soil, food, fruits, vegetables or even medicinal and cosmetic products can harm the human body through inhalation, ingestion or direct contact (Alsherbiny et al., 2019). Ingestion is considered one of the main routes of exposure to poisons. Ingested natural or chemical toxins can injure or impair body functions through damage or death of living tissues (Alnasser et al., 2022; Nasiri et al., 2023). Exposure to toxicants has been regarded to be one of the main contributors to disorders, including autoimmune diseases (Pollard et al., 2010), fatty liver disease (Al-Eryani et al., 2015), neural dysfunction-related disorders such as Alzheimer’s disease and Parkinson’s disease, thyroid and ovarian problems, breast cancer, and obesity (Jamshidi et al., 2020).

Antidotes are therapeutic substances that neutralize the toxic effects of a drug, poison or toxin. Antidotes can mediate various mechanisms, including preventing the absorption and binding of poison, neutralizing poison directly, antagonizing the effect of poison on the end-organ, and inhibiting the transformation of poison into more toxic metabolites (Chacko and Peter, 2019; Aruwa et al., 2020). In recent years, the importance of using herbal antidotes in the management of poisonings has been highlighted. The evidence-based benefits of the phyto-antidotes against adverse chemical agents have also been confirmed (Aruwa et al., 2020; Lysiuk et al., 2020). Different studies have revealed antidote and protective effects on natural or chemical toxicities the following administration of medicinal plants and their main constituents, including turmeric, black cumin, milk thistle, cinnamon, barberry and green tea (Alsherbiny et al., 2019). Additionally, recent studies have emphasized the importance of understanding the molecular mechanisms through which these plants exert their antidotal effects, which could further validate their use in modern medicine. The concept of antidotes has a long history in traditional remedies and different substances of plant, animal, and mineral origin were used as antidotes (Magowska, 2021).

In the traditional Persian medicine (PM) textbooks, many materia medica have been introduced called “tiryaq” (equivalent of antidote in current nomenclature), which have played an important role in the treatment of some diseases and ailments, including poisoning (Al-ahwazi, 2007; Avicenna, 2009). Tiryāq is a therapeutic agent that has a plant, mineral or animal origin or a combination of these substances. Meanwhile, medicinal plants have always played an important role as antidotes (Sonneville et al., 2011; Bénéchet et al., 2019). The primary role of the tiryāq in the response to poisoning is to neutralize the toxic effects of toxicants on the human body and normally to create immunity (Aleem et al., 2020). Moreover, it was used for other medical purposes, including management of chronic cough, stomach discomfort, respiratory problems, fever, colic, some neurologic ailments, and so on (Sonneville et al., 2011; Bénéchet et al., 2019; Aleem et al., 2020; Taghizadieh et al., 2020). Tiryāq strengthens the main organs such as the heart (Al-ahwazi, 2007). Other uses of tiryāqs include prophylaxis in epidemics (Ansari et al., 2020; Mahroozade et al., 2021), antioxidant effects (Khan et al., 2020) and stimulating the immune system (Nigar and Itrat, 2013; Khan et al., 2020).

In traditional medicine, the antidote properties of medicinal plants against all kinds of poisons have been mentioned, however, they have been less investigated and identified in pharmacological studies. Many pharmacological studies still lack sufficient scientific rigor or fail to explore the full therapeutic potential of these plants. This study aims to introduce some medicinal plants with antidote properties and their medical uses, according to the main textbooks of traditional PM. Furthermore, this review aims to critically analyze the existing evidence on the pharmacological properties of these plants and discuss the potential gaps in the current research landscape.

## 2 Methods

In this study, we surveyed the most famous textbooks of ancient traditional PM, including *Al-Qānūn fī al-Ṭibb* (The Canon of Medicine) by Avicenna (Bénéchet et al., 2019) and the most comprehensive pharmacopeia, namely, *Makhzan al-Adwīyah* by Aghīlī Shīrazī (Sonneville et al., 2011), *Al-Shamil fī al-Ṣina*ʿ*a al-Ṭibbīyah* by Ibn al-Nafīs (Nikhat and Fazil, 2023), *Tuḥfah al-Muminīn* by Muḥammad Mumin Tunkabunī (Salehi et al., 2018), *Qarābādīn Kabīr* by Aghīlī Shīrazī (Aghili Khorasani, 1970) to find all mentioned medicinal plants with tiryāq properties, their used parts, and their traditional uses and effects. Then, scientific names of the plants were obtained using herbal textbooks. The search terms were the scientific names and common names of each plant combined with “antidote,” “teryāgh,” “tiryāq,” “taryāq,” “teryāghiat,” “pādzahr,” “cardioprotective,” hepatoprotective,” “neuroprotective,” “immunomodulatory” “antioxidant,” “anti-inflammatory,” “antibacterial,” “antiviral” and “antifungal,” “antidote,” “detoxification.” To this end, electronic databases, including PubMed, Scopus, Google Scholar, Web of Science and other relevant ones were searched for studies describing ethnomedicinal uses and pharmacological and phytochemical aspects of the medicinal plant. Data were collected from May 1980 up to May 2023. Only published articles in English language were included in this review. Finally, we collected and classified the items related to the subject.

## 3 Potential therapeutic roles of antidotal plants

Tiryāq has been widely used in traditional medicine, and one of the most important of them is the antidote of various poisons and the treatment of poisoning (Nikhat and Fazil, 2023; Aghili Khorasani, 2009; Avicenna, 2009). Poison refers to any edible or drinkable substance that is not compatible with human life and after entering the body affects it and as a result causes human fatality by destroying the innate heat (Avicenna, 2009; Tabib Gilani, 2015). The antidote can protect the body against poison/toxic substances that disturb its equilibrium state, as well as expel them that enter the body. Moreover, other applications of an antidote included potential effects in the prevention and even treatment of disorders related to human exposure to contaminated food and air toxic agents (Aghili Khorasani, 2009; Avicenna, 2009). In the traditional PM, various antidotes have been mentioned that have been used individually or in combination. The antidotes act on poisons and remove them from the body or protect against the effects of poisons on the body. The antidote has protective effects on various organs of the human body, including the principal organs (brain, heart, and liver) and strengthens them (Aghili Khorasani, 2009; Avicenna, 2009). Avicenna believed that an antidote is any medicine that has the ability to maintain the strength and health of the soul and through it, the soul repels the harmful effects of the poison (Vaghasloo et al., 2017; Bénéchet et al., 2019; Nasiri et al., 2023). Traditional data on medicinal plants with antidote properties recommended in PM are shown in Table 1. Moreover, their pharmacological activities are summarized in Table 2 and their antidote and detoxification activities are described in Table 3. In this section, we selected ten plants, which people use in abundance such as fennel, nigella, citron, apple, saffron, ajwain, pistachio, garlic, coriander, and ginger. The biological activities of their main phytochemicals in related studies are also discussed.

**TABLE 1:** Medicinal plant with antidote (tiryāq) properties in Persian medicine (PM).

Scientific name	Common name	Traditional names	Used part	Brain tonic	Heart tonic	Liver tonic	Exhilarant	Deobstruent (Mufattiḥ)	Strength of natural heat	Antidotal activity as per PM literature
*Allium sativum* L.	Garlic	*Sīr/thum*	Bulb				+	+	+	Beneficial for the bite of vipers, all kinds of snakes, and scorpions Useful for tarantula, insects, rabid dog’s bites, and snake venom
*Anethum graveolens* L.	Dill	*Shibat/shiwīd*	Leaves, seeds					+		Antidotal property
*Alpinia officinarum* Hance	Lesser galangal	*Khūlanjān*	Root			+		+	+	Antidotal property
*Aristolochia clematitis* L.	Birthworts	*Zarāwand*	Root					+		Useful for insect bites, Scorpion sting
*Berberis vulgaris* L.	Barberry	*Zirishk/ambarbārīs*	Fruit, root		+	+	+			Antidote to deadly poisons Viper bite antidote
*Capparis spinosa* L.	Caper	*Cabir*	Fruit					+	+	Orally or topically, it is an antidote to most poisons
*Cinnamomum camphora* (L.) J.Presl	Camphor	*Kāfūr*	Resin/gum	+	+		+			Antidote for hot poison and scorpion poison, insect repellent
*Citrus aurantium* L.	Bitter orange	*Nārinj*	Fruit, peel	+			+			Antidote activity for insect bites and scorpion stings
*Citrus limon* (L.) Osbeck	Lemon	*Līmū*	Fruit		+	+	+	+		Antidote activity for insect bites
*Citrus medica* L.	Citron	*Turanj*	Fruit	+	+	+			+	Antidote activity for snake, viper and scorpion stings
*Coriandrum sativum* L.	Coriander	*Gishnīz/Kuzburah*	Fruit		+		+			Antidotal property
*Crocus sativus* L.	Saffron	*Zaʿfarān*	Stigma		+	+	+	+	+	Antidotal property
*Curcuma zedoaria (Christm.)* Roscoe	Jadwar	*Jadwār*	Root	+	+	+	+	+	+	Antidote for all kinds of poisons, snake, viper, tarantula, and scorpion bites
*Doronicum grandiflorum* Lam.	Leopard’s bane	*Durūnaj*	Root	+	+	+	+	+	+	Antidote for stings of scorpions, tarantulas and other poisonous animals
*Syzygium aromaticum* (L.) Merr. & L.M.Perry	Clove	*Mīkhak/qaranful*	Fruit	+	+	+	+	+	+	Antidotal property
*Foeniculum vulgare* L.	Fennel	*Rāzyānaj*	Fruit				+	+	+	Antidote for scorpion venom, insect stings, bee stings and dog bites
*Laurus nobilis* L.	Laurel	*Ghar*	Fruit	+			+	+		Antidotal activity for all poisons, antidote for snake and scorpion venom and bee stings, insects repellent
*Lavandula angustifolia* Mill.	Lavender	*Usṭūkhuddūs*	Flower	+	+		+	+	+	Antidotal property
*Malus domestica* Borkh.	Apple	*Sīb/tuffaḥ*	Fruit	+	+	+	+		+	Antidote to poisons, especially scorpion venom, improver of poisonous drugs
*Matricaria chamomilla* L.	Chamomile	*Bābūneh*	Flower	+		+		+		Antidotal activity, insects repellent
*Mentha spicata* L.	Spearmint	*Na’na’*	Aerial parts		+		+		+	Antidote for scorpion venom and dog bites
*Myrtus communis* L.	Myrtle	*Ās, Mūrd*	Fruit, leaf		+				+	Scorpion and tarantula venom antidote
*Nigella sativa* L.	Black cumin/black seeds	*Sīyah-dāneh/shunīz*	Seed					+	+	Antidote for tarantula bites and dog bites
*Pimpinella anisum* L.	Anise	*Anīsūn*	Seed	+	+	+		+		Antidotal property, Insect repellent
*Pistacia vera* L.	Pistachio	*Pisteh/fustuq*	Fruit	+	+	+	+	+	+	Antidote activity for insect bites and scorpion stings
*Pyrus communis* L.	Pear	*Gulābī/kumasra*	Fruit		+		+		+	Antidotal property
*Rheum palmatum* L.	Chinese rhubarb	*Raivand*	Root		+	+	+	+		Antidote activity for insect bites and scorpion stings
*Tanacetum parthenium* (L.) Sch. Bip.	Feverfew	Uqḥuwān	Flower					+		Antidotal property
*Trachyspermum ammi* (L.) Sprague.	Ajwain	*Zinīyān*	Seed					+	+	Antidote activity for insect bites and scorpion stings
*Zataria multiflora* Boiss.	Shirazi Thyme	Āwīshan	Leaves	+				+		Antidote activity for insect bites and scorpion stings
*Zingiber officinale* Roscoe	Ginger	Zanjabīl	Rhizome	+		+			+	Antidote activity for insect bites

**TABLE 2:** Pharmacological activities of medicinal plants with antidote (tiryāq) properties in PM.

Scientific name	Family	Common name	Antidotal activity/detoxification	Antioxidant activity	Anti-inflammatory activity	Immunomodulatory effect	Antibacterial	Antifungal	Antiviral	Hepatoprotective	Neuroprotective	Cardio protective	References
*Allium sativum* L.	*Amaryllidaceae*	Garlic	+	+	+	+	+	+	+	+	+	+	Bhandari (2012), Hoseinifard et al. (2018), Li et al. (2018), El-Saber Batiha et al. (2020), Rouf et al. (2020)
*Anethum graveolens* L.	*Apiaceae*	Dill	+	+	+	+	+	+	+	+	−	+	Ajarem et al. (2021), Amalraj et al. (2022), Li and Wang (2022), Noumi et al. (2023)
*Alpinia officinarum* Hance	*Zingiberaceae*	Lesser galangal	−	+	+	+	+	+	+	+	+	−	Liu et al. (2020), Koçak and Yılmaz (2022), Ni et al. (2022), Hu et al. (2023)
*Aristolochia clematitis* L.	*Aristolochiaceae*	Birthworts	−	+	+	+	+	+	−	−	−	−	Bartha et al. (2019), Moroz et al. (2021), Kumar and Yadav (2022)
*Berberis vulgaris* L.	*Berberidaceae*	Barberry	+	+	+	+	+	+	+	+	+	+	Kalmarzi et al. (2019), Emamat et al. (2020), El-Zahar et al. (2022)
*Capparis spinosa* L.	*Capparaceae*	Caper	−	+	+	+	−	−	+	+	−	+	Mousavi et al. (2016), Kalantari et al. (2018)
*Cinnamomum camphora* (L.) J.Presl	*Lauraceae*	Camphor	−	+	+	+	+	+	−	−	−	−	Chen et al. (2020), Xiao et al. (2021), Luo et al. (2022), Sobhy et al. (2023)
*Citrus aurantium* L.	*Rutaceae*	Bitter orange	−	+	+	+	+	+	+	+	+	+	Shen et al. (2017), Shu et al. (2020)
*Citrus limon* (L.) Osbeck	*Rutaceae*	Lemon	−	+	+	+	+	+	+	+	+	+	Klimek-Szczykutowicz et al. (2020), Khan et al. (2022), Yadav et al. (2022)
*Citrus medica* L.	*Rutaceae*	Citron	−	+	+	+	+	+	+	+	+	+	Al-Yahya et al. (2013), Peng et al. (2019), Pooja et al. (2021), El Hawary et al. (2022)
*Coriandrum sativum* L.	*Apiaceae*	Coriander	−	+	+	+	+	+	+	+	+	+	Kačániová et al. (2020), Dėnė et al. (2021), Foudah et al. (2021), Perera et al. (2022), Atrooz et al. (2023)
*Crocus sativus* L.	*Iridaceae*	Saffron	+	+	+	+	+	+	+	+	+	+	Rezaee-Khorasany et al. (2019), Jafari-Sales and Pashazadeh (2020), Zhong et al. (2020), Ouahhoud et al. (2021)
*Curcuma zedoaria (Christm.)* Roscoe	*Zingiberaceae*	Jadwar *(white turmeric)*	−	+	+	+	+	+	+	+	+	+	Mahmoudi et al. (2020), Amrullah and Fachrial (2021), Sari and Ginting (2022)
*Doronicum grandiflorum Lam.*	*Asteraceae*	Leopard’s bane	−	+	+	−	−	−	−	−	−	−	Badalamenti et al. (2021)
*Syzygium aromaticum* (L.) Merr. and L.M. Perry	*Myrtaceae*	Clove	+	+	+	+	+	+	+	+	−	−	Rajan and Surya (2019), Amir Rawa et al. (2022), Devkota et al. (2022), Elbestawy et al. (2023)
*Foeniculum vulgare* L.	*Apiaceae*	Fennel	−	+	+	+	+	+	+	+	+	+	Orhan et al. (2012), Belabdelli et al. (2020), Sfar et al. (2023)
*Laurus nobilis* L.	*Rosaceae*	Laurel	+	+	+	+	+	+	+	+	+	+	Ravindran et al. (2013), Chbili et al. (2020), Khodja et al. (2021)
*Lavandula angustifolia* Mill.	*Lamiaceae*	Lavender	−	+	+	+	+	+	−	−	+	+	Andrys et al. (2017), Pandur et al. (2021)
*Malus domestica* Borkh.	*Rosaceae*	Apple	+	+	+	+	+	−	−	+	+	+	Lu et al. (2019), Lee et al. (2020b), Wang et al. (2023)
*Matricaria chamomilla* L.	*Asteraceae*	Chamomile	+	+	+	+	+	+	−	+	+	+	Mehrim et al. (2006), Stanojevic et al. (2016), AlTamimi et al. (2020), Asadi et al. (2020)
*Mentha spicata* L.	*Lamiaceae*	Spearmint	+	+	+	−	+	+	+	+	+	−	Saleem et al. (2000), Kehili et al. (2020), Oueslati et al. (2020)
*Myrtus communis* L.	*Myrtaceae*	Myrtle	+	+	+	+	+	+	+	+	+	+	Aykac et al. (2019), Özbeyli et al. (2020), Roozitalab et al. (2022)
*Nigella sativa* L.	*Ranunculaceae*	Black seeds	+	+	+	+	+	+	+	+	+	+	Yaman et al. (2010), Yesmin et al. (2013), Attia et al. (2020), Abd-Elkareem et al. (2021), Esharkawy et al. (2022), Ojueromi et al. (2022)
*Pimpinella anisum* L.	*Apiaceae*	Anise	+	+	+	+	+	+	+	+	+	+	Pontes et al. (2019), Ghlissi et al. (2020), El-Moslemany et al. (2023)
*Pistacia vera* L.	*Anacardiaceae*	Pistachio	−	+	+	−	+	+	+	+	+	+	Azadedel et al. (2021), Haleshi et al. (2022)
*Pyrus communis* L.	*Rosaceae*	Pear	+	+	+	−	−	−	+	+	+	+	Kolniak-Ostek et al. (2020), Lee et al. (2020a), Anser et al. (2021), Singh et al. (2021)
*Rheum palmatum* L.	*Polygonaceae*	Chinese rhubarb	+	+	+	+	−	+	+	+	+	−	Gao et al. (2016), Li et al. (2019), Park et al., 2020; Bhat (2021)
*Tanacetum parthenium* (L.) Sch.Bip.	*Asteraceae*	Feverfew	−	+	+	+	+	−	+	−	+	+	Recinella et al. (2020), Pooja et al. (2021)
*Trachyspermum ammi* (L.) Sprague.	*Apiaceae*	Ajwain	+	+	+	+	+	+	+	+	+	+	Velazhahan et al. (2010), Sharma et al. (2018), Timalsina et al. (2023)
*Zataria multiflora* Boiss	*Lamiaceae*	Shirazi Thyme	+	+	+	+	+	+	+	+	+	−	Karimi and Meiners (2021), Ahmadi et al. (2022), Arab et al. (2022)
*Zingiber officinale* Roscoe	*Zingiberaceae*	Ginger	+	+	+	+	+	+	+	+	+	+	Fahmi et al. (2019), Kaushik et al. (2020), Nerilo et al. (2020), Abdi et al. (2021), Arcusa et al. (2022), Harun and Mohamad (2022), Yousuf et al. (2023)

**TABLE 3:** Antidote and detoxification activities of medicinal plants in current studies.

Plant	Study Type	Antidotal Activity	Dose Range	Model Used	Controls	Duration	Extract Type	Detoxification Mechanisms	Properties	Ref
*Allium sativum* L.	*In vivo*	Zinc	3 mg/L (Zinc sulfate) + 0.5–2.5% garlic in diet	Goldfish (*Carassius auratus*)	Untreated control	45 days	Garlic (dietary)	Garlic reduces zinc accumulation in tissues, indicating antioxidant properties	Hepatoprotective, antioxidant, anti-inflammatory	Hoseinifard et al. (2018)
	*In vivo*	Nicotine	50 mg/kg/day (AMSO2)	Rats	Dexamethasone (1 mg/kg/day)	21 days	AMSO2	Regulation of p38 MAPK, Nrf-2 and Bcl-2/Bax signaling pathways AMSO2 reverses apoptosis and oxidative stress induced by cigarette smoke	Hepatoprotective, antioxidant, anti-inflammatory	Li et al. (2018), Ehnert et al. (2012)
*Anethum graveolens* L.	*In vitro*	Paracetamol	100 mg/kg (AG1), 200 mg/kg (AG2)	Mice	Isotonic NaCl	7 days	Aqueous extract	AG protects against liver acute toxication induced by paracetamol	Hepatoprotective, antioxidant, antimicrobial	Korkmaz et al. (2021)
*Berberis vulgaris* L.	*In vivo*	Lipopolysaccharide	10 mg/kg (Berberine)	Mouse calvarial model	PBS	5–7 days	-	Berberine blocks LPS-induced osteoclast recruitment and bone resorption	Hepatoprotective, nephroprotective, cardioprotective, neuroprotective	Zhou et al. (2012)
	*In* *vivo*	Cholera toxin	-	Rabbit model	-	-	-	Berberine inhibits secretory response of enterotoxins in the intestinal loop model Effective as chloramphenicol or tetracycline in controlling experimental cholera		Sack et al. (1982), Dutta et al. (1972)
*Crocus sativus* L.	*In vivo*	Snake venom	5 g/kg (ethanol) + 40–160 mg/kg (Saffron)	Rats	Distilled water	4 weeks	Aqueous extract	Saffron protects against ethanol-induced toxicity, improving biochemical markers and reducing apoptosis	Hepatoprotective, neuroprotective, antioxidant	Razavi and Hosseinzadeh (2015)
*Crocus sativus* L.	*In vivo*	Ethanol	5 g/kg	Rats	Distilled water	4 weeks	Aqueous extract	Reducing lipid peroxidation, increasing GSH content, diminishing histopathological damages, moderating specific inflammatory biomarkers (TNF-α and IL-6), inhibiting apoptosis by attenuating Bax/Bcl2 ratio (both mRNA and protein) and decreasing the levels of caspase-3, caspase-8, and caspase-9	Hepatoprotective, neuroprotective, antioxidant	Rezaee-Khorasany et al. (2019)
*Laurus nobilis* L.	*In vivo*	Paracetamol	200 mg/kg and 400 mg/kg	Rats	Silymarin (25 mg/kg)	-	Methanol extract	Protects liver against paracetamol-induced injury, reducing serum enzyme levels	Hepatoprotective, anti-inflammatory, antioxidant	Ravindran et al. (2013)
*Lavandula angustifolia* Mill.	*In vitro*	Sitophilus oryzae	0.25–6 mg/cm^2^	-	-	48 h	Essential oil	Essential oils show insecticidal effects on S. oryzae (lavandulyl)	Neuroprotective, antioxidant, anti-inflammatory	Al-Harbi et al. (2021)
*Malus domestica* Borkh.	*In vivo*	Fusarium toxins	100 ppb (Aflatoxin B1)	Tilapia fish	-	14 weeks	-	Dietary ginger alleviates aflatoxicosis symptoms in tilapia	Neuroprotective, anti-venom	Gutzwiller et al. (2007)
*Matricaria chamomilla* L.	*In vivo*	Aflatoxin B1	100 ppb	Tilapia fish	-	14 weeks	-	Ginger showed the best detoxifying effects on aflatoxin	Hepatoprotective, neuroprotective, anti-inflammatory	Mehrim et al. (2006)
*Mentha spicata* L.	*In vivo*	Benzoyl peroxide	10–20 mg/kg	Mice	-	1 h before BPO treatment	Spearmint extract	Protects against BPO-induced oxidative stress and enhances DNA synthesis	Hepatoprotective, antioxidant, anti-inflammatory	Saleem et al. (2000)
*Myrtus communis* L.	*In vivo* and *in vitro*	P. falciparum	10 mg/kg	Mice	-	4 days	Methanolic extract	Significant suppression of parasitemia; further studies needed on mechanisms	Hepatoprotective, neuroprotective, antioxidant	Naghibi et al. (2013)
*Nigella sativa* L.	*In vivo*	Gentamicin	100 mg/kg (Gentamicin) + 0.2–0.4 mL/kg (Nigella)	Rats	Control group	-	Nigella sativa seed	Reduces nephrotoxicity and restores antioxidant enzyme activity	Hepatoprotective, nephroprotective, antioxidant	Yaman et al. (2010)
*Nigella sativa* L.	*In vivo*	Lead	0.8% garlic or 2% Nigella	Rabbits	Positive and negative controls	8 weeks	Garlic/Nigella	Enhances growth performance and detoxification of lead		Attia et al. (2020)
*Pimpinella anisum* L.	*In vivo*	Metronidazole	200 mg/kg (anise)	Rats	Saline control	30 days	Anise seed extract	Mitigates neurotoxic effects of metronidazole	Hepatoprotective, neuroprotective, anti-inflammatory	El-Moslemany et al. (2023)
*Pyrus communis* L.	*In vitro*	1-bisphenol A, 2-alcohol	-	Korean pear (Pyrus pyrifolia)	-	-	-	Korean pear stimulates ADH in human liver cells. It also stimulates ALDH activities and decreases blood alcohol	Hepatoprotective, antioxidant, anti-inflammatory	118
*Rheum palmatum* L.	*In vivo*	Mercuric chloride	-	-	-	-	-	Rhubarb tannins could protect rats from hexavalent chromium induced renal intoxication by scavenging hydroxyl radicals	Hepatoprotective, nephroprotective, anti-inflammatory	Gao et al. (2016)
*Syzygium aromaticum (*L*.)* Merr. and L.M. Perry	*In vivo*	Lead and cadmium	-	-	-	-	-	-	Hepatoprotective, antioxidant, antimicrobial	Rajan and Surya (2019)
*Tanacetum parthenium (*L*.)* Sch. Bip.	*In vivo*	-	-	-	-	-	-	Antidote for excessive opium use; unknown mechanism	Neuroprotective, anti-inflammatory, antioxidant	Pareek et al. (2011)
*Trachyspermum ammi* (L.) Sprague.	*In vitro*	Aflatoxins G1	-	-	-	-	-	Modification of lactone ring structure	Anti-inflammatory and hepatoprotective	Bhadra (2020)
*Zataria multiflora* Boiss	*In vivo*	Sodium nitrite	-	-	-	-	-	Anti-oxidative and anti-inflammatory properties; further studies needed to determine protective effects	Hepatoprotective, antioxidant, antimicrobial	Ahmadi et al. (2022)
*Zingiber officinale* Roscoe	*In vivo*	Paracetamol	200 mg/kg and 300 mg/kg	Rats	N-acetylcysteine (500 mg/kg)	24 h	Ethanol extract	Protects against paracetamol-induced hepatotoxicity; effective dose-dependent response	Neuroprotective, hepatoprotective, anti-inflammatory	Malaysia (2004)
*Zingiber officinale* Roscoe	*In vivo*	Cadmium	200 ppm (cadmium)	Rats	Normal chow	6 weeks	Ginger concentrate	Ginger reduces liver cadmium accumulation and serum parameter	Neuroprotective, hepatoprotective, anti-inflammatory	Egwurugwu et al. (2007)

### 3.1 Fennel (*Foeniculum vulgare* L.)

According to the traditional PM, fennel has been used for the treatment of a variety of eye, respiratory, liver and kidney diseases. It has exhilarating properties, and tonic for stomach and eyes. It can strengthen innate heat. Fennel is an antidote to animal poisons and is useful for poisonous animals’ stings such as scorpions, bees and the like. Consumption of fennel decoction is also recommended for rabid dog bites (Aghili Khorasani, 1970; Aghili Khorasani, 2009; Avicenna, 2009). In addition, it has shown protective effects on humor infection and is useful in prolonged fevers (Nikhat and Fazil, 2023). The primary phytoconstituents of this plant have been identified as phenols, phenolic glycosides, and volatile fragrance compounds such as trans-anethole, estragole, and fenchone. Fennel has shown many pharmacological properties, including antifungal, antibacterial, antioxidant, anti-inflammatory, hepatoprotective and neuroprotective (Rahimi and Ardekani, 2012), and antiviral (Orhan et al., 2012) activities. This plant also has immunomodulatory (Darzi et al., 2018), and cardioprotective effects (Natarajan and Grace, 2019). The antioxidant activity of fennel is attributed to phenolic compounds, while the volatile fragrance compounds make it a great flavoring agent (Rather et al., 2016).

### 3.2 Black seed (*Nigella sativa* L.)

*Nigella sativa* L. from the Ranunculaceae family, is considered a widely used medicinal plant all over the world. There are many therapeutic uses of this plant in several traditional medicine systems such as Unani, Ayurveda and Siddha (Ahmad et al., 2013). In traditional PM, black seed is widely used in the treatment of various ailments and diseases. It is considered antidote to cold poisons. Moreover, in combination with lukewarm water, it is useful to treat someone who has been attacked by a rabid dog or tarantula. The smoke from burning black seeds is an effective way to repel insects (Aghili Khorasani, 1970; Aghili Khorasani, 2009). It has been demonstrated that black seed has a wide range of biological activities, including antioxidant, antimutagenic, antidiabetic, analgesic, antibacterial, immunomodulatory, anti-inflammatory, spasmolytic, bronchodilator (Ahmad et al., 2013; Tavakkoli et al., 2017), and antiviral (Esharkawy et al., 2022) properties. The bioactive component, thymoquinone (TQ), present in the essential oil of black seed, is chiefly responsible for these therapeutic effects (Al-Ali et al., 2008).

Numerous animal studies have highlighted the protective effects of black seed and TQ on the heart (Yesmin et al., 2013), brain, lung, kidney and liver have been shown against some toxic agents either natural or chemical toxins (Ahmad et al., 2013; Tavakkoli et al., 2017). Moreover, preclinical research has documented its potential as an antidote for various poisoning, including mycotoxins, endotoxins, metals (Al, lead, mercury, and cadmium), pesticides (imidacloprid, propoxur, acetamiprid, chlorpyrifos, fenitrothion), solvents (ethanol, CCl_4_, toluene), and environmental pollutants (such as Bisphenol A). However, human studies are still needed to confirm the effectiveness of black seed as an antidote against human poisoning (Tavakkoli et al., 2017).

### 3.3 Citron (*Citrus medica* L.)

The *citron* (*Citrus medica L*.) belongs to the herbal family of Rutaceae. In Unani and Ayurvedic medicine, citron has been used for therapeutic purposes, the effects of its peel and whole fruit have been mentioned to improve flatulence, and strengthen the stomach and heart (Al-Yahya et al., 2013). Based on traditional PM textbooks, this plant is a heart, liver and brain tonic (Aghili Khorasani, 1970; Aghili Khorasani, 2009; Avicenna, 2009). Moreover, the *citron* has been introduced as an effective antidote to every kind of poison. Its consumption together with honey repels the harm of all poisons. Drinking citron extract has been used as an antidote against snake bites in humans. Citron oil also has antidote properties. Inhaling citron oil has been considered a remedy for conditions such as air pollution and epidemics (Aghili Khorasani, 2009). The poultice of this plant has been used for scorpion and horned snake bites. Citron seeds are used in all oral poisonings and bites. In all animal poisons, the use of peeled citron seeds orally or as a topical preparation is an alternative to “*Tiryāq Fārūq*” and is stronger than “*Tiryāq Kabīr*.” Moreover, its consumption along with hot water has been experienced in scorpion bites. Anointing with citron seed oil will keep the scorpion away (Aghili Khorasani, 2009).

In Greek texts, the use of different parts of citrus fruits as an antidote for “poison and venom” is also mentioned. Taking citron before meals is an antidote for any type of poison and is recommended orally and topically for scorpion and viper bites. Regarding its efficacy, it has been stated that citron peel perfumes the breath and its scent calms the spirit (Arias and Ramón-Laca, 2005). This plant has antioxidant, anti-inflammatory, antimutagenic, antibacterial, antifungal, antiviral (El Hawary et al., 2022), immunostimulatory (Peng et al., 2019), cardioprotective (Al-Yahya et al., 2013), hepatoprotective, and neuroprotective activities (Pooja et al., 2021). Citron is known as a potent antioxidant due to the presence of bioactive components, mainly phenolics, flavanones, vitamin C, and pectin. The main components of fruit peel oil include iso-limonene, citral, and limonene, and the fruit is rich in vitamins and minerals, especially vitamin A, vitamin C, niacin, and thiamin (Chhikara et al., 2018). In an animal study, methanolic extract of citrus peel showed a protective effect on acute cyanide poisoning-induced seizures and oxidative stress. These neuroprotective effects may be explained by inhibiting the excessive production of oxidative stress as well as maintaining antioxidant defense mechanisms (Abdel Moneim, 2014).

### 3.4 Apple (*Malus domestica* Borkh.)

Apple is one of the nutrient-rich and most popular fruits. This fruit has many benefits for the human body in terms of having a lot of vitamin C, fiber, antioxidants, etc. (Acquavia et al., 2021). From the viewpoint of traditional PM, it is very effective in treating poisons, especially scorpion venom. It also has a protective role in epidemics and air pollution and is an ameliorator of toxic drugs. It is recommended to use apple or its leaf extract for scorpion venom or other poisons, either when drunk or applied externally (Aghili Khorasani, 2009), In addition to the fruit, its leaf extract also has these properties (Avicenna, 2009). Sour apple is astringent, and relieves vomiting and thirst (Aghili Khorasani, 1970; Aghili Khorasani, 2009). This fruit is tonic for the brain, heart and liver, and also has exhilarant properties (Aghili Khorasani, 2009). Apples are highly consumed and nutritious fruit and contain various bioactive compounds, including pectins, dietary fibers, vitamins, oligosaccharides, triterpenic acids, and phenolic compounds. The high antioxidant properties of apples are due to the higher content of these phenolic compounds (Asma et al., 2023). There is now substantial scientific evidence that these bioactive compounds found in apples and their peels have the potential to promote human health by reducing the risk of cardiovascular disease, diabetes mellitus, inflammation, and cancer (Patocka et al., 2020). Some studies have also shown that apple has immunity enhancement, anti-inflammatory, antioxidant, antibacterial, cardioprotective, hepatoprotective (Patocka et al., 2020), and neuroprotective effects (Lu et al., 2019).

### 3.5 Ajwain (*Trachyspermum ammi* (L.) Sprague.)

*Trachyspermum ammi* (L.) Sprague. belongs to Apiaceae family (Zarshenas et al., 2013). Ajwain has been traditionally administered for the management of various ailments such as febrile conditions, cough, respiratory distress, fatigue, neuropathic and chronic pains (Kamalinejad et al., 2021). Ajwain has protective effects against cardiovascular issues, hepatotoxicity, and oxidative stress, showing promise in treating various types of poisoning. In the traditional PM, the antidote potency is explained to Ajwin. Its use is considered an antidote to poisons and insect and animal bites. Consumption of ajwain is useful in eliminating the harms of opium use and also for quitting. A medicinal rinse of ajwain is fast-acting to relieve scorpion venom (Aghili Khorasani, 2009). It is very useful in chronic fevers (Avicenna, 2009). Thymol is the main component in ajwain, which has antiseptic, antifungal, antibacterial, antioxidant, and anti-inflammatory properties. In addition, thymol has the ability to reduce the levels of C-reactive protein (CRP), Interleukin-1 beta (IL-1β), IL-6, tumor necrosis factor-alpha (TNF-α), TNF-β, and matrix metalloproteinase 9 (MMP9) (Korani and Jamshidi, 2020). Administration of dietary ajwain extract in rat models could attenuate the hexachlorocyclohexane-induced oxidative stress and hepatotoxicity (Anilakumar et al., 2009). The aqueous extract of ajwain showed antimicrobial effects on several bacterial strains, including *Enterococcus faecalis*, *Staphylococcus aureus*, *Escherichia coli*, *P. aeruginosa*, *S. typhimurium,* and *Shigella flexneri* (Kaur and Arora, 2009). It has protective effects on the cardiovascular system, including calcium channel blocking effect, which leads to a decrease in heart rate and blood pressure, positive ionotropic and negative chronotropic effects, cholinomimetic effects, which could cause bradycardia, lipid-lowering effect, and protective effect on body weight gain (Boskabady et al., 2014). Antifungal activity of different types of ajwain extract is also documented against *Aspergillus* species, *Epidermophyton floccosum, Microsporum canis, Trichophyton mentagrophytes, Candida albicans, and C. utilis* (Boskabady et al., 2014). Due to the calcium channel blocking effect, ajwain seed extract showed antihypertensive, antispasmodic, bronchodilator and hepatoprotective activities. Oral administration of ajwain in an animal model of liver injury induced by carbon tetrachloride (CCL_4_) and lethal dose of paracetamol (1 g/kg), showed protective effects on paracetamol- and CCL_4_-induced hepatic injuries and reduced liver enzymes (Gilani et al., 2005).

### 3.6 Garlic (*Allium sativum* L.)

Garlic (*Allium sativum* L.) is one of the most widely used medicinal plants for flavor and spice, which has many health benefits. It belongs to the Liliaceae family (Bhandari, 2012; Verma et al., 2023). This medicinal plant and its components have capabilities such as scavenging free radicals, anti-inflammatory, anti-cholesterol, anti-gastric ulcer, anti-bacterial, anti-cancer and antioxidant (Koscielny et al., 1999).

In traditional PM, garlic has antidote and exhilarant properties (Aghili Khorasani, 2009; Avicenna, 2009), and repels damage caused by air pollution, epidemics and incoherent waters (Aghili Khorasani, 2009). The use of large quantities of garlic and wine is of great benefit in the bites of vipers, snakes, and scorpions, to a point where there is almost no equal. If it is applied to the bite site, it is of great benefit by absorbing the poison (Nikhat and Fazil, 2023). Consumption of garlic is also useful for tarantula, insects, rabid dog’s bites, and snake venom. Garlic alone or in combination with fig leaves, cumin, olive oil and wine has been used to absorb all the toxins and relieve the discomfort associated with them (Aghili Khorasani, 2009).

Garlic consists of sulfur-containing plant compounds such as alliin, allicin, ajoenes, vinyldithiins, and flavonoid compounds such as quercetin. Extracts and isolated compounds of garlic have shown various pharmacological activities, including antibacterial, antiviral, antifungal, antioxidant, anti-inflammatory, and anticancer, antidiabetic, antihypertensive, anti-obesity, and antithrombotic activities (El-Saber Batiha et al., 2020). According to the available evidence, garlic can be considered an antidote or protective plant against many toxic agents. This plant has the ability to modulate the activity of several metabolizing enzymes. A number of *in vitro* and animal pre-clinical studies showed protective effects of garlic and its major components against cardiotoxicity, hematotoxicity, neurotoxicity, hepatotoxicity, nephrotoxicity, intestinal toxicity, pulmonary toxicity, bone marrow toxicity, and reproductive toxicity/teratogenicity induced by natural and chemical agents. The protective effect of garlic against natural and chemical toxins is attributed to its various properties, including free radical scavenging, antioxidant effect, reduction of lipid peroxidation, anti-inflammatory effect, a chelating agent in heavy metal poisoning, cytoprotective activities, increased protein production in damaged tissues, and suppression of apoptosis (Dorrigiv et al., 2020). Phospholipase A2 (PLA2) enzyme is one of the major components of snake venom. The antidotal effects of garlic against snake venom have been demonstrated through inhibition or inactivation of the PLA2 enzyme (Asad et al., 2013; Asad et al., 2014).

### 3.7 Pistachio (*Pistacia vera* L.)

Pistachio belongs to the Anacardiaceae family. In traditional PM, pistachio has been used for different ailments. It is considered a mind, brain, heart, liver and stomach tonic. Consuming pistachio alone or with wine is useful for getting rid of scorpion poisons and other insect bites (Aghili Khorasani, 2009). Especially, the decoction of pistachio in wine is very beneficial for insect bites (Avicenna, 2009). In other words, Pistachio is an antidote to insect bites and poisons (Tonekaboni, 2007). Also, consuming pistachio along with sugar improves the side effects of air pollution (Aghili Khorasani, 2009). Based on modern phytotherapy, it has antioxidant, anti-inflammatory, cardioprotective (Ersöz, 2023), hepatoprotective (Iranmanesh et al., 2017), antimicrobial, antiviral, antifungal and neuroprotective activities (Mandalari et al., 2021). Pistachio is considered a rich source of protein, fiber, monounsaturated fatty acids, minerals (copper, manganese, potassium, phosphorous, chromium, magnesium, iron, zinc, and selenium) and vitamins (vitamin B6, thiamin, vitamins E,riboflavin, and folate) as well as carotenoids, phenolic acids, flavonoids and anthocyanins (Mandalari et al., 2021).

Pistachio kernel contains antioxidants such as tocopherols, phylloquinone, carotenoids, chlorophyll and flavonoids, therefore pistachio is among the foods with high total antioxidant capacity (Liu et al., 2014). Some of the beneficial properties of pistachio can be partially related to its antioxidant metabolites (Gentile et al., 2007). In an animal study investigating the cardioprotective effect of pistachio skin extract, it was shown that this extract has antioxidant activity and can reduce apoptosis and deoxyribonucleic acid damage in heart damage (Ersöz, 2023). A recent systematic review and meta-analysis showed that pistachio has a beneficial effect on some cardiometabolic risk factors. Based on the findings of 11 clinical trials, pistachio consumption significantly reduced blood glucose (FBS and HbA1C), serum lipids, systolic blood pressure, and inflammatory biomarkers (Ghanavati et al., 2020). In an animal study, it was shown that treatment with pistachio supplementation eliminated spatial memory disorders due to neurotoxicity of cisplatin and vincristine, and improved cognitive and motor functions (Golchin et al., 2015). Other animal studies have shown the neuroprotective potential of pistachio, including reducing anxiety-like behavior, improving memory, cognitive and motor impairments, and increasing working memory and physical power (Mandalari et al., 2021). Pistachio had shown bactericidal effects on a wide range of Gram-positive bacteria, including *Listeria* monocytogenes, *S. aureus* and methicillin-resistant clinical isolates of *S. aureus* (MRSA) (Bisignano et al., 2013). Chronic gavage of pistachio hydro-alcoholic extract showed hepatoprotective activity against experimentally induced liver damage by CCL_4_ in rats. Treatment of different doses of pistachio extract decreased AST and ALT levels as well as plasma LDL concentration (Iranmanesh et al., 2017). Polyphenols from pistachio showed anti-herpetic effects on herpes simplex virus type 1 (HSV-1). Treatment with polyphenol-rich extracts of natural shelled (NPRE) pistachio kernels could reduce the expression of the viral proteins and viral DNA synthesis (Musarra-Pizzo et al., 2020).

### 3.8 Saffron *(Crocus sativus* L.)

According to the traditional PM, saffron has an exhilarant property and is a tonic for heart and liver and innate heat (Aghili Khorasani, 2009; Avicenna, 2009). In addition, it was the reformer of phlegm infection and prevents and protects it from change and corruption (Aghili Khorasani, 1970; Aghili Khorasani, 2009). Saffron, obtained from the dried stigma of *Crocus sativus* L is used as a spice, coloring and flavoring agent in foods and cosmetics worldwide (Xing et al., 2021). Biologically active metabolites present in this plant such as carotenoids, flavonoids, terpenoids, amino acids, glycosides, starch, mineral matter and alkaloids have shown potential medicinal properties (Bukhari et al., 2018; Xing et al., 2021; Abu-Izneid et al., 2022). In modern pharmacological studies, these components exhibited several properties such as anti-inflammatory, antioxidant, anxiolytic (Bukhari et al., 2018), antiviral, antitumor, antianxiety, hypoglycemic, hypolipidemic, memory-enhancing, immunomodulatory (Xing et al., 2021), antidepressant, antitussive, chelating metal, antifungal (Ouahhoud et al., 2021), cardioprotective (Bukhari et al., 2018; Xing et al., 2021), neuroprotective (Xing et al., 2021), hepatoprotective (Ouahhoud et al., 2021), and antibacterial (Naim et al., 2022) activities. Saffron and its active metabolites have demonstrated cardiac protective effects through modulating oxidative stress, inflammation, blood pressure, lipid and glycemic profiles or direct anti-atherogenic effects (Kadoglou et al., 2021). Saffron’s antioxidant activity, largely attributed to safranal, crocin, and carotene, plays a major role in its protective effects against oxidative stress, inflammation, and cardiovascular diseases (Bukhari et al., 2018; Abu-Izneid et al., 2022). The protective effects of phenolic metabolites in saffron petal extract against bacteria that potentially cause food-borne diseases were evaluated. Based on the findings, these phenolic compounds showed antibacterial effects on *S. aureus*, *Salmonella typhimurium*, *E. coli* and *Listeria monocytogenes* (Naim et al., 2022).

### 3.9 Coriander *(Coriandrum sativum* L.)

Coriander is a popular medicinal plant that belongs to Apiaceae family. It has several biological activities, including antioxidant, hypoglycemic, hypolipidemic, analgesic, anti-inflammatory, anti-cancer, immunomodulatory (Laribi et al., 2015), antiviral (Perera et al., 2022) antifungal, antibacterial, hepatoprotective and lead-detoxifying effects (Sahib et al., 2013). Moreover, neuroprotective effects such as anxiolytic, sedative-hypnotic and anticonvulsant activities were reported for the seeds and leaves of the plant (Jakubczyk et al., 2020). Several cardiovascular benefits of this botanical drug such as antihypertensive, anti-atherogenic, antiarrhythmic as well as cardioprotective effects have been mentioned (Mahleyuddin et al., 2021). In PM, Coriander has exhilarant properties and is a brain and heart tonic (Aghili Khorasani, 1970; Aghili Khorasani, 2009). Coriander volatile metabolites were evaluated for *in vitro* and *in vivo* antioxidant activity and hepatoprotective effects on carbon tetrachloride damage. *In vitro* antioxidant activity as free radical scavenging capacity, as inhibitory activity of essential oils on 2,2-diphenyl-1-picrylhydrazyl (DPPH) and OH radicals and effects on lipid peroxidation (LP) was measured. Its essential oils could reduce stable DPPH radicals in a dose-dependent manner and neutralize H_2_O_2_ (Pandey et al., 2011). The essential oil and aqueous extract of coriander leaves showed inhibitory activity against *Bacillus subtilis*, *S. aureus*, *Klebsiella pneumonia* and a pathogenic fungus, *C. albicans* (Foudah et al., 2021).

### 3.10 Ginger *(Zingiber officinale* Roscoe)

Ginger belongs to the Zingiberaceae family (Mao et al., 2019). According to traditional PM, ginger strengthens memory, digestion, stomach, and it is liver and brain tonic (Aghili Khorasani, 1970; Aghili Khorasani, 2009). In addition, this plant strengthens the natural heat (Aghili Khorasani, 2009). Ginger is also useful for removing animal toxins (Aghili Khorasani, 1970; Aghili Khorasani, 2009) and insect bites (Avicenna, 2009).

Accumulated studies have revealed that ginger indicates many biological activities, such as antibacterial (Sebiomo et al., 2011), antifungal (Nerilo et al., 2020), antiviral (Kaushik et al., 2020), immunomodulatory (Harun and Mohamad, 2022), anti-inflammatory (Abdi et al., 2021), antioxidant activity, cardiovascular protective (Abdi et al., 2021), neuroprotective (Yousuf et al., 2023), and hepatoprotective effects (Fahmi et al., 2019). Long-term studies have shown that ginger and its various active compounds have anti-inflammatory activity. It was initially suggested that the anti-inflammatory activity of ginger is mainly related to its inhibitory effect on prostaglandin and leukotriene synthesis. Both fresh ginger (consisting mainly of gingerols) and dried ginger extracts (the main source of shogaols) inhibit lipopolysaccharide (LPS)-induced prostaglandin E2 (PGE2) production (Ozkur et al., 2022). Phenolic metabolites in ginger, such as gingerol, have a protective effect on ischemic heart disease in rats (Dissanayake et al., 2020). Ginger plant extract can modulate immune responses that intensify the inflammation process at the cellular level. The active components of this plant have antioxidant activity and have effects similar to non-steroidal anti-inflammatory drugs and stop the metabolism of arachidonic acid by inhibiting cyclooxygenase and lipoxygenase pathways (Dissanayake et al., 2020). A botanical drug derived from fresh ginger rhizome has been proven to have an antiviral effect on human respiratory syncytial virus (HRSV) infection by reducing HRSV-induced plaque formation in respiratory mucosal cell lines. Therefore, high concentrations of ginger can stimulate mucosal cells to secrete IFN-β, which helps fight viral infections by reducing virus adhesion and internalization. This effect is very useful in the management of colds and fever with mucous secretions and the management of complications from cough and asthmatic conditions. Lyophilized water extract of ginger has an antiviral effect on hepatitis C virus infection. In a specific study, ginger has been proven to inhibit viral replication within HepG2 cells infected with hepatitis C virus by affecting viral RNA, and another study detailed that ginger is effective in reducing these cells. Hepatitis C virus loads, α-fetoprotein level and markers related to liver function such as aspartate aminotransferase (AST) and alanine aminotransferase (ALT) are prescribed in HCV patients (Dissanayake et al., 2020).

## 4 Future perspectives

The high toxicity of various modern chemicals and their pervasive potency to pollute the environment and diffuse within the human body necessitate effective preventive measures against acute and chronic poisoning. One such approach is the investigation of antitoxins derived from natural sources, especially medicinal plants, traditionally utilized for therapeutic purposes across various cultures.

In this regard, Sardari et al. investigated the therapeutic properties of caper plant, which is one of the types of antidotes. The caper plant, whose scientific name is *Capparis spinosa,* is one of the prominent examples of anti-toxic food. This plant affects a wide range of different body systems, including the digestive system, nervous system, respiratory system, spleen, and reproductive system. It can serve as an antidote for most poisons (Sardari et al., 2018). “Tiryaq Arba” has been introduced as a polyherbal formulation that has the potential to prevent acute respiratory infections. They showed that it has antiviral properties against different viruses, including coronavirus, adenovirus, respiratory syncytial virus, and para-influenza virus. Additionally, this antidote is an excellent medicine in epidemics. It is one of the heart- and brain-tonic and protective drugs. It is also used as a solvent and antidote in acute respiratory infections and is a potential prophylactic drug used for COVID-19 (Ansari et al., 2020). In another study, Khan et al. introduced Tiryaq-e-Wabai for prophylaxis during cholera, plague, and other epidemics, which also has antioxidant and immune system-stimulating effects. The properties of the plants that make up this antidote have already been investigated in an animal model, and their antiviral, antitussive, and expectorant activities are a solid basis for the use of prophylaxis in COVID-19 (Khan et al., 2020). Kalam et al. investigated the various therapeutic effects of Tiryaq-e-Wabai compounds and stated that Tiryaq-e-Wabai was used as an antidote, prophylaxis, anti-epidemic, and poisoning caused by bites (Kalam et al., 2020).

Emami et al. examined the opinions of Iranian medical sages about the nutrition of the elderly, and stated that an antidote is one of the recommended foods or medicines in PM, which is made from abundant amounts of natural substances and strengthens the heart and stomach, senses and stimulates the appetite (Emami et al., 2014). In a study conducted by Shamsi-Baghbanan et al., liver-protecting plants from the point of view of Ibn Sina and introduced Teriyaq Kabir as one of the combined drugs that protect the liver (Shamsi-Baghbanan et al., 2014). In another study conducted by Nigar and Itrat at the Greek Medicine Hospital in Bangalore, the effectiveness of an antidote as an immune system stimulant in the elderly (Nigar and Itrat, 2013).

Similarly, *Teriyaq Kabir* has been highlighted for its liver-protective properties (Emami et al., 2014). Clinical trials have further substantiated the immune-boosting potential of these antidotes, particularly in vulnerable populations such as the elderly (Nigar and Itrat, 2013). The present study investigated the mechanisms of medicinal plants with possible beneficial effects on poisoning and related organs based on the suggestions of PM. Key pathways include modulation of NF-κB and Nrf2 signaling, balancing pro-inflammatory and anti-inflammatory cytokines, and activating enzymatic and non-enzymatic antioxidant defenses. These mechanisms provide broad-spectrum protection across various tissues, consistent with PM’s holistic view that treatments should enhance overall health rather than targeting isolated organs (Bahramsoltani and Rahimi, 2020). For example, the role of pro-inflammatory cytokines (IL-1β, IL-6, and TNF-α) is well-documented in poisoning cases. Exposure to toxins like lead and carbon monoxide can induce chronic inflammation, oxidative stress, and damage to organs like the liver. Medicinal plants counteract these effects by reducing inflammation and oxidative stress, as demonstrated in studies on compounds like garlic polysaccharides, which suppress the expression of inflammatory factors (Feldmann et al., 2020; Shao et al., 2020).

The primary advantages of plant-based antidotes include their affordability, broad-spectrum efficacy, and minimal side effects (Lysiuk et al., 2020; Kenari et al., 2021). Edible medicinal plants, such as apple, lemon, saffron, pear, and pistachio, are particularly noteworthy for their safety, antioxidant properties, and ability to modulate the immune system. These plants also exhibit protective effects on vital organs such as the brain, heart, and liver through mechanisms such as reducing inflammation and enhancing cellular defense pathways. Their widespread daily use underscores their potential for therapeutic application. Emerging evidence highlights the potential of specific plants for targeted therapies. For instance, pistachio’s cardioprotective and neuroprotective activities are linked to its rich antioxidant composition (Ersöz et al., 2023). Active components of saffron, e.g., crocin and safranal, are effective in modulating oxidative stress and inflammation, as well as protecting against cardiovascular and neurological damage (Xing et al., 2021; Abu-Izneid et al., 2022). Similarly, ginger’s bioactive compounds inhibit inflammatory pathways and show antiviral effects on hepatitis C and respiratory syncytial viruses (Dissanayake et al., 2020).

The protective effects of these plants align with PM’s concept of enhancing the body’s “instinctive heat” or internal force as a means to combat illness. By stimulating cellular defense mechanisms, these plants not only prevent damage but also promote recovery, offering a comprehensive strategy for managing poisoning and its complications. The study’s limitations consist of a reliance on preclinical evidence, variability in plant preparation methods, studies with small sample sizes, limited demographic diversity, and the lack of standardized, placebo-controlled clinical trials in some studies. Additionally, long-term safety and efficacy data are insufficient, emphasizing the need for rigorous future research to validate these findings.

## 5 Conclusion

Medicinal plants suggest a promising attitude for developing safe and effective antidotes, indicating a natural resource in modern pharmacotherapy. Although traditional and contemporary evidence underscores their therapeutic potential, it is essential to address the existing methodological and translational gaps to fully shed light into their beneficial and detrimental effects. Future research that integrates the holistic principles of phytomedicine with rigorous scientific methodologies may revolutionize the management of poisoning and related circumstances. Also, systematic investigations into these plants and their active metabolites should be prioritized to identify novel therapeutic agents. Such studies may provide a deeper understanding of the pharmacological properties of these botanicals, paving the way for therapeutic innovations. Clinical trials are critical to validating the efficacy and safety profiles of these compounds, ensuring that they meet the rigorous standards required for therapeutic use. Additionally, mechanistic studies will further elucidate the molecular targets and pathways involved, enhancing our understanding of how these natural products exert their therapeutic effects. By bridging the gap between traditional and modern medicine, we can unravel the full potential of medicinal plants, ultimately resulting in improved health outcomes and novel strategies for managing poisoning and other related conditions. The integration of interdisciplinary approaches will be essential in advancing this field, fostering collaborations between ethnobotanists, pharmacologists, and clinicians to translate these findings into clinical practice.
